# Association of N-butylphthalide treatment with 1-year activities-of-daily-living recovery in convalescent ischemic stroke patients with chronic disease burden: a post-marketing real-world non-randomized controlled study

**DOI:** 10.3389/fmed.2026.1905628

**Published:** 2026-08-03

**Authors:** Xiaobing Tian, Xiyu Zhao, Lifeng Wang, Hongyan Lu, Juan Hao, Zhiquan Liu, Chenxi Fan, Xianjia Ning, Yan Li

**Affiliations:** 1 Department of Neurology, Tianjin Medical University General Hospital, Tianjin, China; 2 Department of Critical Care Medicine, Tianjin Jizhou People's Hospital, Tianjin, China; 3 Department of Nursing, Tianjin Jizhou People's Hospital, Tianjin, China; 4 College of Pharmacy, Nankai University, Tianjin, China; 5 Institute of Clinical Epidemiology and Evidence-Based Medicine, Tianjin Jizhou People's Hospital, Tianjin, China; 6 Laboratory of Epidemiology, Tianjin Neurological Institute, Tianjin, China; 7 Tianjin Neurological Institute, Key Laboratory of Post-Neuroinjury Neuro-Repair and Regeneration in Central Nervous System, Ministry of Education and Tianjin City, Tianjin, China

**Keywords:** activities of daily living, Barthel Index, chronic disease burden, ischemic stroke, n-butylphthalide

## Abstract

**Background and objective:**

ADL recovery is essential for maintaining independence and reducing care burden after IS, particularly in patients with chronic vascular-metabolic disease burden. Although NBP has been studied for acute treatment, recurrence prevention, and cognitive protection, its association with ADL recovery in convalescent IS remains unclear. This study evaluated whether NBP treatment was associated with 1-year ADL recovery in a post-marketing real-world non-randomized controlled study.

**Methods:**

This was a post-marketing, real-world, prospective, non-randomized controlled study conducted in Jizhou District, Tianjin, China. Patients received either NBP plus basic treatment or basic treatment alone and were followed for 12 months. The primary outcome was change in BI score from baseline to 1 year. Secondary outcomes included change in mRS score, mRS >2, BI ≤ 60, and item-level BI changes. Chronic disease burden was defined as the cumulative number of hypertension, diabetes, hyperlipidemia, obesity, and coronary heart disease. Multivariable regression, subgroup analyses, IPTW analysis, and conservative death-imputation analysis were performed. This post-marketing, real-world, prospective, non-randomized controlled study was registered in the Chinese Clinical Trial Registry (ChiCTR2000039118).

**Results:**

Among 1,190 enrolled patients, 1,095 completed 1-year functional assessment, including 533 in the NBP group and 562 in the control group. Patients receiving NBP had slightly worse baseline mRS scores and a higher chronic disease burden than controls. At 1 year, the NBP group showed greater BI improvement than the control group (1.31 ± 13.99 vs. −1.11 ± 15.56; *P* = 0.0067), whereas change in mRS did not differ significantly (−0.18 ± 0.96 vs. −0.17 ± 1.05; *P* = 0.877). Item-level analyses showed greater improvements in grooming, toilet use, feeding, transfers, mobility on level surfaces, dressing, and stair climbing. In the fully adjusted model, NBP treatment remained associated with greater BI improvement (β = 2.40, 95% CI: 0.77–4.03; *P* = 0.0038), but not with mRS change. NBP was also associated with lower adjusted odds of ADL dependency at 1 year. The association with BI improvement remained significant in IPTW and conservative death-imputation analyses.

**Conclusion:**

In this post-marketing real-world non-randomized controlled study, NBP treatment was associated with better 1-year ADL recovery in convalescent IS patients with chronic disease burden, mainly reflected by BI improvement and self-care or mobility-related gains rather than global disability change. These findings support ADL-centered assessment in long-term post-stroke care and suggest that functional recovery should be considered alongside recurrent event prevention and cognitive protection in aging stroke populations.

**Clinical Trial Registration:**

[ChiCTR2000039118]; http://www.chictr.org.cn.

## 1 Introduction

Stroke remains one of the most serious neurological disorders worldwide and is a leading cause of death, disability, and long-term dependence. According to the Global Burden of Disease Study 2021, stroke accounted for approximately 7.3 million deaths and 160.5 million disability-adjusted life-years globally in 2021, ranking among the major contributors to death and disability worldwide (1). The World Stroke Organization further emphasized that the absolute burden of stroke has continued to increase over recent decades, with substantial growth in incident stroke, prevalent stroke, stroke-related deaths, and disability-adjusted life-years from 1990 to 2021 (2). In China, the burden is particularly prominent. In 2019, there were approximately 3.94 million incident stroke cases, of which 2.87 million were ischemic stroke, indicating that IS remains the dominant stroke subtype and a major public health challenge (3). With improvements in acute stroke care and survival, the clinical focus has gradually expanded from preventing death and recurrence to preserving independence and reducing long-term disability. For stroke survivors, impairment in activities of daily living is closely related to loss of independence, caregiver burden, institutional care, reduced quality of life, and increased health-care utilization. This issue is especially important among older adults and patients with chronic disease burden, in whom vascular-metabolic comorbidities such as hypertension, diabetes, hyperlipidemia, obesity, and coronary heart disease may compromise functional recovery and accelerate frailty-related decline.

In recent years, increasing attention has been paid to long-term functional recovery after IS, particularly in real-world populations with multimorbidity. Conventional secondary prevention strategies, including antiplatelet therapy, lipid-lowering treatment, blood pressure control, glucose management, lifestyle modification, and rehabilitation, have reduced recurrent vascular events and improved prognosis. However, many patients continue to experience persistent limitations in basic self-care and mobility. BI is widely used to assess basic activities of daily living after stroke, covering domains such as feeding, grooming, toilet use, transfers, mobility, dressing, stair climbing, bathing, and continence (4). Compared with global disability scales, BI may be more sensitive to changes in daily functional independence, particularly in community-dwelling stroke survivors with mild-to-moderate disability. Therefore, evaluating BI change provides clinically meaningful information on whether a treatment is associated with preservation or recovery of practical daily living capacity.

NBP has attracted increasing interest as a multitarget neuroprotective agent for IS. Experimental and clinical studies suggest that NBP may exert neuroprotective effects through several mechanisms, including improving microcirculation, protecting mitochondrial function, reducing oxidative stress and neuroinflammation, inhibiting neuronal apoptosis, and promoting collateral circulation and neurovascular repair (5, 6). Clinical evidence has also accumulated. A randomized, double-blind trial showed that 90-day administration of DL-3-NBP improved functional outcomes in patients with acute IS (7). More recently, a multicenter randomized clinical trial reported that butylphthalide was safe and effective in patients with acute IS receiving reperfusion therapy (8). An updated systematic review and meta-analysis of randomized controlled trials further suggested that NBP treatment was associated with improved neurological deficits and increased BI scores in patients with acute IS, although evidence regarding long-term death or dependency remained insufficient (9). These findings support the potential role of NBP in stroke recovery, but most available studies have focused on the acute or early subacute stage and have mainly assessed short-term neurological improvement or global disability.

Several important uncertainties remain. First, the evidence for NBP in the convalescent stage of IS is still limited, particularly in community-based real-world settings where patients often have a high burden of chronic vascular-metabolic diseases. Second, previous studies have tended to focus on recurrent stroke, mRS, neurological deficit scores, or cognitive outcomes, whereas ADL recovery as a primary functional endpoint has been less well characterized. Third, multimorbidity and chronic disease burden are increasingly recognized as key determinants of functional decline and frailty in older adults, but few studies have examined whether the association between NBP treatment and post-stroke functional recovery persists in patients with substantial chronic disease burden. Finally, although previous work from our study showed that NBP was associated with a lower risk of recurrent IS and total stroke events over 12 months, functional outcomes assessed by mRS and BI did not differ significantly between treatment groups in that analysis (10). Subsequent studies from the same research platform suggested that NBP may help prevent post-stroke cognitive decline, and that its cognitive benefits may vary according to diabetes status (11, 12). However, whether NBP is associated with 1-year recovery of basic activities of daily living in convalescent IS patients, especially in the context of chronic disease burden, remains unclear.

Therefore, the present study aimed to evaluate the association between NBP treatment and 1-year ADL recovery in convalescent IS patients using data from post-marketing real-world non-randomized controlled study. The primary outcome was the 1-year change in BI score. Secondary outcomes included changes in mRS, unfavorable functional outcome, BI item-level changes, and subgroup analyses according to age, sex, diabetes, obesity, baseline functional status, and chronic disease burden. By focusing on ADL recovery rather than only recurrent vascular events or global disability, this study seeks to provide clinically relevant evidence for long-term functional management of stroke survivors with chronic disease burden.

## 2 Methods

### 2.1 Study design and setting

This was a post-marketing, real-world, prospective, non-randomized controlled study conducted in Jizhou District, Tianjin, China between April 2021 and May 2024. The study was designed to evaluate the association between NBP treatment and 1-year ADL recovery among patients with convalescent IS. Participants were recruited from a community-based stroke management program and followed for 12 months after enrollment. The study was reported in accordance with CONSORT principles as applicable to non-randomized, real-world controlled studies (13).

The study was conducted in accordance with the Declaration of Helsinki (14) and was approved by the Ethics Committee of Tianjin Medical University General Hospital (IRB2020-YX-056-02). Written informed consent was obtained from all participants or their legal representatives before enrollment. The study was registered in the Chinese Clinical Trial Registry (ChiCTR2000039118).

### 2.2 Study population

Eligible participants were permanent residents of Jizhou District aged 18 years or older who had been diagnosed with non-cardioembolic IS within the previous 6 months. The diagnosis was confirmed according to clinical manifestations and neuroimaging findings. Patients were enrolled during the convalescent stage rather than the acute phase, and all participants were required to be capable of self-care or to have a stable caregiver who could assist with medication administration and follow-up assessment.

The exclusion criteria were as follows: history of hemorrhagic stroke; malignant tumor; coagulation disorder or platelet count < 100 × 10^9^/L; severe hepatic or renal dysfunction; severe cardiac dysfunction; pregnancy; participation in other clinical trials; severe aphasia or cognitive/communication impairment that precluded reliable assessment; or inability to provide informed consent. Patients without complete baseline and 1-year BI or mRS assessments were excluded from the primary functional recovery analysis.

### 2.3 Treatment grouping

Participants were assigned to the NBP group or the control group using a non-randomized, sequential (staged) enrollment design implemented at the community level. Allocation was determined by the recruitment phase during which a participant was enrolled rather than by randomization or by individual clinical judgement. Patients enrolled in the first recruitment phase received NBP plus basic treatment, whereas patients enrolled in the second phase received basic treatment alone.

Patients in the NBP group received NBP soft capsules at a dose of 0.2 g 3 times daily for 12 months, in addition to basic treatment. Basic treatment included antiplatelet agents, lipid-lowering drugs, antihypertensive medications, antidiabetic therapy, and other guideline-based treatments according to individual clinical indications. Patients in the control group received basic treatment without NBP. To enhance adherence, medication instructions were provided at enrollment, NBP was dispensed regularly, and empty medication bottles were collected during follow-up visits.

### 2.4 Baseline data collection

Baseline information was collected through face-to-face interviews, physical examinations, laboratory tests, and medical record review by trained investigators using standardized procedures. Demographic variables included age, sex, and years of education. Lifestyle factors included smoking status and alcohol use. Medical history included hypertension, diabetes, hyperlipidemia, coronary heart disease, and previous stroke-related information.

Physical measurements included height, weight, systolic blood pressure, and diastolic blood pressure. BMI was calculated as weight in kilograms divided by height in meters squared. According to Chinese adult BMI criteria, participants were categorized as underweight, normal weight, overweight, or obese (15). Laboratory tests were performed after an overnight fast of at least 12 h and included fasting blood glucose, glycated hemoglobin, total cholesterol, triglycerides, high-density lipoprotein cholesterol, low-density lipoprotein cholesterol, homocysteine, and high-sensitivity C-reactive protein.

Baseline functional status was assessed using mRS and BI. The mRS was used to evaluate global disability, whereas BI was used to assess basic ADL. BI item-level scores included bowel control, bladder control, grooming, toilet use, feeding, transfers, mobility on level surfaces, dressing, stair climbing, and bathing.

### 2.5 Assessment of chronic disease burden

To reflect the vascular-metabolic multimorbidity profile of the study population, a chronic disease burden score was constructed using five common chronic conditions: hypertension, diabetes, hyperlipidemia, obesity, and coronary heart disease. Each condition was assigned 1 point, yielding a total score ranging from 0 to 5. For descriptive and subgroup analyses, chronic disease burden was further categorized as low burden, moderate burden, and high burden, defined as 0–1, 2, and ≥3 conditions, respectively.

Hypertension was defined as a self-reported physician diagnosis, current use of antihypertensive medication, or elevated blood pressure measured at baseline. Diabetes was defined as a self-reported physician diagnosis, current use of glucose-lowering medication, or laboratory evidence of hyperglycemia. Hyperlipidemia was defined as a self-reported physician diagnosis, current use of lipid-lowering medication, or abnormal lipid profile. Obesity was defined according to Chinese BMI criteria. Coronary heart disease was defined according to self-reported history or medical records.

### 2.6 Follow-up and outcome assessment

Participants were followed for 12 months after enrollment. Follow-up assessments were conducted by trained investigators through community visits, outpatient review, telephone contact, and medical record verification when necessary. Information on medication use, recurrent vascular events, death, and functional status was collected during follow-up.

The primary outcome was 1-year ADL recovery, measured by the change in BI score from baseline to 1 year. The change in BI score was calculated as:

1-year BI score minus baseline BI score. A positive value indicated improvement in ADL, whereas a negative value indicated functional decline.

Secondary outcomes included the 1-year change in mRS score, unfavorable functional outcome, dependency in ADL, and changes in individual BI components. The change in mRS score was calculated as 1-year mRS score minus baseline mRS score. A negative value indicated improvement in global disability. Unfavorable functional outcome was defined as mRS >2 at 1 year, and dependency in ADL was defined as BI ≤ 60 at 1 year. BI item-level changes were calculated using the same approach as the total BI score.

### 2.7 Statistical analysis

Continuous variables were summarized as mean and standard deviation or median and interquartile range, depending on distribution. Categorical variables were expressed as numbers and percentages. Baseline characteristics were compared between the NBP and control groups using Student's *t*-test or the Mann–Whitney U test for continuous variables and the chi-square test or Fisher's exact test for categorical variables, as appropriate.

The primary analysis evaluated the association between NBP treatment and 1-year change in BI score. Multivariable linear regression models were constructed using ΔBI as the dependent variable and NBP treatment as the primary independent variable. A stepwise modeling strategy was used to examine the robustness of the association. Model 1 was unadjusted. Model 2 adjusted for age, sex, and education. Model 3 further adjusted for lifestyle factors and vascular-metabolic comorbidities, including smoking status, alcohol use, BMI, hypertension, diabetes, hyperlipidemia, and coronary heart disease. Model 4 additionally adjusted for baseline mRS, baseline BI, systolic blood pressure, diastolic blood pressure, low-density lipoprotein cholesterol, homocysteine, and high-sensitivity C-reactive protein. Regression coefficients, 95% confidence intervals, and *P* values were reported.

Secondary analyses were performed for ΔmRS, unfavorable functional outcome, dependency in ADL, and BI item-level changes. Linear regression was used for continuous functional change outcomes. Logistic regression was used for binary outcomes, including mRS >2 and BI ≤ 60 at 1 year. Because mRS is an ordinal disability scale, analyses of ΔmRS were interpreted as secondary and exploratory, with emphasis placed on BI as the primary ADL outcome.

Subgroup analyses were conducted to explore whether the association between NBP treatment and ΔBI differed by age group, sex, diabetes status, obesity status, baseline BI status, and chronic disease burden. Interaction terms between NBP treatment and each subgroup variable were included in the regression models to calculate *P* values for interaction. These analyses were considered exploratory and were interpreted cautiously.

To reduce potential treatment-selection bias inherent in a post-marketing real-world non-randomized controlled study design, propensity score methods were used in sensitivity analyses (16). The propensity score was estimated using baseline covariates that were clinically relevant or imbalanced between treatment groups, including demographic characteristics, lifestyle factors, vascular-metabolic comorbidities, baseline functional status, blood pressure, and laboratory indicators. Inverse probability of treatment weighting (IPTW) was applied to create a weighted pseudo-population with improved covariate balance between the NBP and control groups (17). Weighted regression models were then used to re-estimate the association between NBP treatment and ΔBI. Covariate balance after weighting was assessed using standardized mean differences.

An additional conservative sensitivity analysis was performed to account for deaths during follow-up. In this analysis, patients who died before the 1-year functional assessment were assigned the worst functional values, with BI set to 0 and mRS set to 6. The primary regression model was then repeated to examine whether the association between NBP treatment and ADL recovery remained consistent. The primary analysis employed a complete case analysis method. To further evaluate the potential impact of incomplete 1-year functional data, we conducted multiple imputation sensitivity analyses for both functional outcomes. The 1-year Barthel Index and modified Rankin Scale scores were estimated using a chain equation-based multiple imputation method based on predicted mean matching.

All statistical tests were two-sided, and *P* values < 0.05 were considered statistically significant. Statistical analyses were performed using SPSS version 25.0 and R software.

## 3 Results

**Figure 1:**
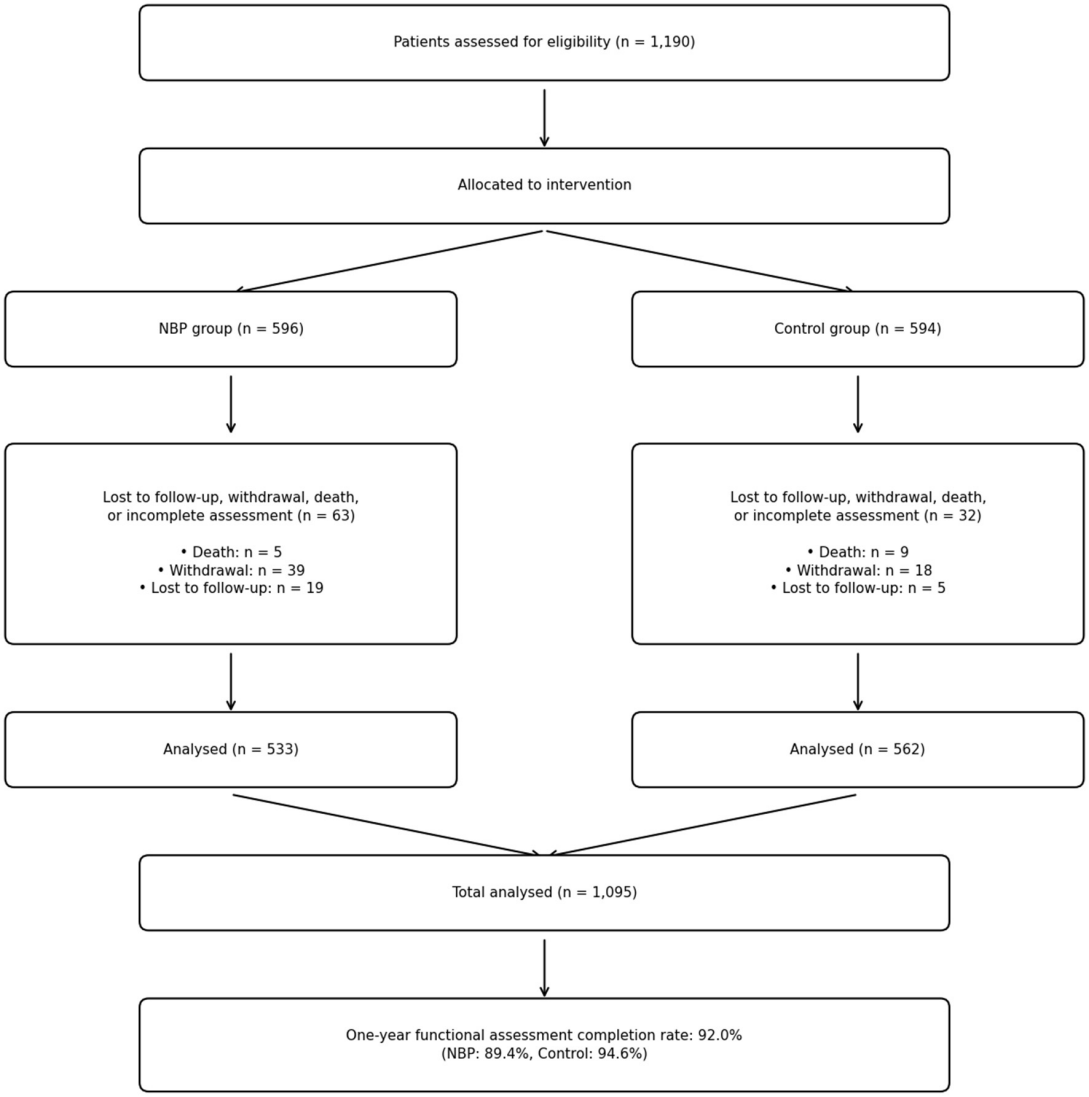
Flow diagram of participant screening, allocation, and 1-year follow-up. This Figure shows the number of patients assessed for eligibility, allocated to the N-butylphthalide (NBP) and control groups by staged, non-randomized sequential community enrollment, and analyzed at 1 year. Reasons for exclusion are shown separately for each group within the exclusion boxes: in the NBP group, death (*n* = 5), withdrawal (*n* = 39), and loss to follow-up (*n* = 19); in the control group, death (*n* = 9), withdrawal (*n* = 18), and loss to follow-up (*n* = 5). Completion rates were 89.4% and 94.6% in the NBP and control groups, respectively.

A total of 1,190 patients with convalescent IS were enrolled in the baseline participants, including 596 patients in the NBP group and 594 patients in the control group. During the 12-month follow-up period, 95 patients were not included in the primary functional recovery analysis because of loss to follow-up, withdrawal, death, or incomplete 1-year functional assessment. Finally, 1,095 patients with complete baseline and 1-year BI and mRS data were included in the main analysis, including 533 in the NBP group and 562 in the control group. The overall completion rate for 1-year functional assessment was 92.0% (Figure 1).

### 3.1 Baseline characteristics and chronic disease burden

The mean age was similar between the NBP and control groups (62.22 ± 8.66 years vs. 62.61 ± 8.27 years, *P* = 0.456). No significant between-group differences were observed in education years, BMI, DBP, diabetes, coronary heart disease, obesity, or baseline BI score. The proportion of men was lower in the NBP group than in the control group (64.4% vs. 70.8%, *P* = 0.026).

Several baseline differences were observed between the two groups. Patients in the NBP group had lower SBP than those in the control group (152.41 ± 20.84 mmHg vs. 156.48 ± 20.87 mmHg, *P* = 0.001), but had a higher prevalence of hyperlipidemia (46.9% vs. 39.1%, *P* = 0.011). Baseline global disability was slightly worse in the NBP group, as indicated by a higher baseline mRS score (1.29 ± 1.21 vs. 1.06 ± 1.11, *P* = 0.001). The baseline BI score was numerically lower in the NBP group, but the difference was not statistically significant (93.74 ± 16.96 vs. 95.20 ± 14.85, *P* = 0.130).

The study population had a substantial burden of vascular-metabolic chronic diseases. The mean chronic disease burden score was higher in the NBP group than in the control group (2.06 ± 1.12 vs. 1.84 ± 1.14, *P* = 0.002). The proportion of patients with high chronic disease burden was also greater in the NBP group than in the control group (34.9% vs. 27.2%), whereas the proportion with low burden was lower (32.5% vs. 41.3%; *P* = 0.004; Table 1).

**Table 1:** Baseline characteristics of patients included in the 1-year functional recovery analysis.

Characteristic	NBP group (*n* = 533)	Control group (*n* = 562)	*P* value
Age, years	62.22 (8.66)	62.61 (8.27)	0.456
Men	343 (64.4)	398 (70.8)	0.026
Education, years	6.70 (3.61)	6.83 (3.53)	0.544
BMI, kg/m^2^	26.67 (3.87)	26.46 (3.25)	0.326
SBP, mmHg	152.41 (20.84)	156.48 (20.87)	0.001
DBP, mmHg	94.60 (11.52)	94.74 (11.61)	0.852
Hypertension	416 (78.0)	409 (72.8)	0.051
Diabetes	183 (34.3)	168 (29.9)	0.131
Hyperlipidemia	250 (46.9)	220 (39.1)	0.011
Coronary heart disease	73 (13.7)	70 (12.5)	0.604
Obesity	175 (32.8)	169 (30.1)	0.358
Baseline mRS score	1.29 (1.21)	1.06 (1.11)	0.001
Baseline BI score	93.74 (16.96)	95.20 (14.85)	0.130
Chronic disease burden score	2.06 (1.12)	1.84 (1.14)	0.002
Chronic disease burden category			0.004
Low burden (0–1 condition)	173 (32.5)	232 (41.3)	
Moderate burden (2 conditions)	174 (32.6)	177 (31.5)	
High burden (>=3 conditions)	186 (34.9)	153 (27.2)	

Values are mean (SD) or *n* (%) unless otherwise indicated. Chronic disease burden score was calculated as the cumulative number of hypertension, diabetes, hyperlipidemia, obesity, and coronary heart disease. BMI, body mass index; SBP, systolic blood pressure; DBP, diastolic blood pressure. Continuous variables were compared using Student's *t*-test (or the Mann–Whitney *U*-test for non-normally distributed variables), and categorical variables were compared using the chi-square test (or Fisher's exact test when expected cell counts were < 5).

Compared with included participants, excluded participants had a worse baseline functional status (mean baseline mRS 1.64 vs. 1.17, *P* = 0.004; mean baseline Barthel Index 86.05 vs. 94.49, *P* = 0.004) and were more frequently allocated to the NBP group (66.3% vs. 48.7%, *P* = 0.001), whereas age, sex, education, body mass index, blood pressure, major comorbidities, and chronic disease burden did not differ significantly (Supplementary Table 1).

### 3.2 One-year changes in ADL and global disability

One-year functional outcomes according to treatment group are presented in Table 2. Patients receiving NBP showed an average increase in BI score of 1.31 ± 13.99 points, whereas patients in the control group showed an average decrease of 1.11 ± 15.56 points (*P* = 0.0067). The between-group difference in BI change was approximately 2.43 points, indicating that NBP treatment was associated with better preservation or recovery of ADL over 1 year.

**Table 2:** One-year functional outcomes according to treatment group.

Outcome	NBP group (*n* = 533)	Control group (*n* = 562)	*P* value
BI score at 1 year	95.06 (15.68)	94.09 (17.40)	0.335
Change in BI score	1.31 (13.99)	−1.11 (15.56)	0.0067
mRS score at 1 year	1.10 (1.13)	0.88 (1.22)	0.002
Change in mRS score	−0.18 (0.96)	−0.17 (1.05)	0.877
mRS >2 at 1 year	58 (10.9)	59 (10.5)	0.914
BI < =60 at 1 year	30 (5.6)	40 (7.1)	0.377

Values are mean (SD) or *n* (%). Change in BI score was calculated as 1-year BI score minus baseline BI score; a positive value indicates improvement. Change in mRS score was calculated as 1-year mRS score minus baseline mRS score; a negative value indicates improvement.

In contrast, changes in global disability measured by mRS did not differ significantly between groups. The mean change in mRS score was −0.18 ± 0.96 in the NBP group and −0.17 ± 1.05 in the control group (*P* = 0.877). Although the 1-year mRS score was higher in the NBP group than in the control group (1.10 ± 1.13 vs. 0.88 ± 1.22, *P* = 0.002), this difference should be interpreted in the context of the higher baseline mRS score in the NBP group. The proportions of patients with mRS >2 at 1 year were similar between the NBP and control groups (10.9% vs. 10.5%, *P* = 0.914). The proportion of patients with BI ≤ 60 at 1 year was numerically lower in the NBP group, but the unadjusted between-group difference was not statistically significant (5.6% vs. 7.1%, *P* = 0.377).

### 3.3 Item-level changes in BI components

Compared with the control group, the NBP group showed significantly greater improvements in several self-care and mobility-related components, including grooming (0.12 ± 1.28 vs. −0.12 ± 1.35, *P* = 0.0028), toilet use (0.23 ± 1.93 vs. −0.15 ± 2.09, *P* = 0.0020), feeding (0.13 ± 1.59 vs. −0.16 ± 1.88, *P* = 0.0056), transfers (0.23 ± 2.28 vs. −0.10 ± 2.52, *P* = 0.026), mobility on level surfaces (0.15 ± 2.43 vs. −0.21 ± 2.61, *P* = 0.017), dressing (0.17 ± 2.27 vs. −0.18 ± 2.15, *P* = 0.0095), and stair climbing (0.16 ± 2.40 vs. −0.21 ± 2.45, *P* = 0.011). No significant between-group differences were observed for bowel control, bladder control, or bathing (Supplementary Table 2).

### 3.4 Multivariable association between NBP treatment and 1-year BI recovery

In the unadjusted model, NBP treatment was associated with greater improvement in BI score (β = 2.43, 95% CI: 0.67 to 4.18; *P* = 0.0069). This association remained statistically significant after adjustment for age, sex, and education (β = 2.36, 95% CI: 0.59 to 4.12; *P* = 0.0089). After further adjustment for smoking, alcohol use, BMI, hypertension, diabetes, hyperlipidemia, and coronary heart disease, NBP treatment remained associated with greater BI improvement (β = 2.56, 95% CI: 0.77 to 4.34; *P* = 0.0050). In the fully adjusted model, which additionally included baseline mRS, baseline BI, SBP, DBP, LDL-C, homocysteine, and hs-CRP, the association remained significant (β = 2.40, 95% CI: 0.77 to 4.03; *P* = 0.0038).

By contrast, NBP treatment was not significantly associated with change in mRS score in any model. In the fully adjusted model, the association between NBP treatment and change in mRS score remained non-significant (β = 0.081, 95% CI: −0.030 to 0.192; *P* = 0.152; Table 3).

**Table 3:** Multivariable association between NBP treatment and 1-year changes in BI and mRS scores.

Outcome and model	β (95% CI)	*P* value
15.5-7.4,13.5242pt**Change in BI score**	
Model 1	2.43 (0.67 to 4.18)	0.0069
Model 2	2.36 (0.59 to 4.12)	0.0089
Model 3	2.56 (0.77 to 4.34)	0.0050
15.6-7.2,-1242ptModel 4	2.40 (0.77 to 4.03)	0.0038
Change in mRS score:	
Model 1	−0.009 (−0.129 to 0.110)	0.878
Model 2	−0.006 (−0.125 to 0.114)	0.928
Model 3	−0.004 (−0.126 to 0.118)	0.948
Model 4	0.081 (−0.030 to 0.192)	0.152

Linear regression models used NBP treatment as the primary independent variable. The control group was the reference group. LDL-C, low-density lipoprotein cholesterol; hs-CRP, high-sensitivity C-reactive protein. Model 1: unadjusted; Model 2: adjusted by age, sex, and education; Model 3: adjusted by Model 2+ smoking, alcohol use, BMI, hypertension, diabetes, hyperlipidemia, and coronary heart disease; Model 4: adjusted by Model 3+ baseline mRS, baseline BI, SBP, DBP, LDL-C, homocysteine, and hs-CRP.

### 3.5 Binary functional outcomes

In the unadjusted analysis, NBP treatment was not significantly associated with mRS >2 at 1 year (OR = 1.04, 95% CI: 0.71 to 1.53; *P* = 0.837). After full adjustment, the association remained statistically non-significant, although the point estimate was directionally favorable (OR = 0.63, 95% CI: 0.37 to 1.07; *P* = 0.086).

For ADL dependency defined as BI ≤ 60 at 1 year, the unadjusted association was not statistically significant (OR = 0.78, 95% CI: 0.48 to 1.27; *P* = 0.315). However, after full adjustment, NBP treatment was associated with a lower odds of ADL dependency at 1 year (OR = 0.42, 95% CI: 0.22 to 0.80; *P* = 0.008; Supplementary Table 3).

### 3.6 Subgroup analyses

In age-stratified analyses, NBP treatment was associated with greater BI improvement in patients aged < 60 years (β = 2.62, 95% CI: 0.57 to 4.67; *P* = 0.012) and showed a non-significant trend in those aged 60–75 years (β = 1.68, 95% CI: −0.56 to 3.92; *P* = 0.141), whereas no association was observed in those aged >75 years (β = 0.84, 95% CI: −28.79 to 30.48; *P* = 0.953). There was no significant interaction by age group (P for interaction = 0.430). Similar findings were observed in men (β = 1.79, 95% CI: 0.02 to 3.56; *P* = 0.048) and women (β = 3.61, 95% CI: 0.11 to 7.11; *P* = 0.043), with no significant interaction by sex (P for interaction = 0.256).

When stratified by diabetes status, the association was significant among patients without diabetes (β = 2.10, 95% CI: 0.41 to 3.78; *P* = 0.015) and directionally favorable but non-significant among those with diabetes (β = 3.05, 95% CI: −0.58 to 6.68; *P* = 0.099). The interaction by diabetes status was not statistically significant (P for interaction = 0.459). In analyses stratified by obesity status, NBP treatment was significantly associated with greater BI improvement among non-obese patients (β = 3.66, 95% CI: 1.74 to 5.59; *P* < 0.001), whereas no significant association was observed among obese patients (β = −0.04, 95% CI: −3.23 to 3.15; *P* = 0.981). The interaction by obesity status was borderline but did not reach statistical significance (P for interaction = 0.068).

Across chronic disease burden strata, the direction of association was consistently favorable. The β values were 2.48 (95% CI: 0.25 to 4.72; *P* = 0.029) in patients with low burden, 2.57 (95% CI: −0.32 to 5.47; *P* = 0.081) in those with moderate burden, and 2.35 (95% CI: −1.25 to 5.94; *P* = 0.200) in those with high burden. There was no significant interaction between NBP treatment and chronic disease burden (P for interaction = 0.943).

**Figure 2:**
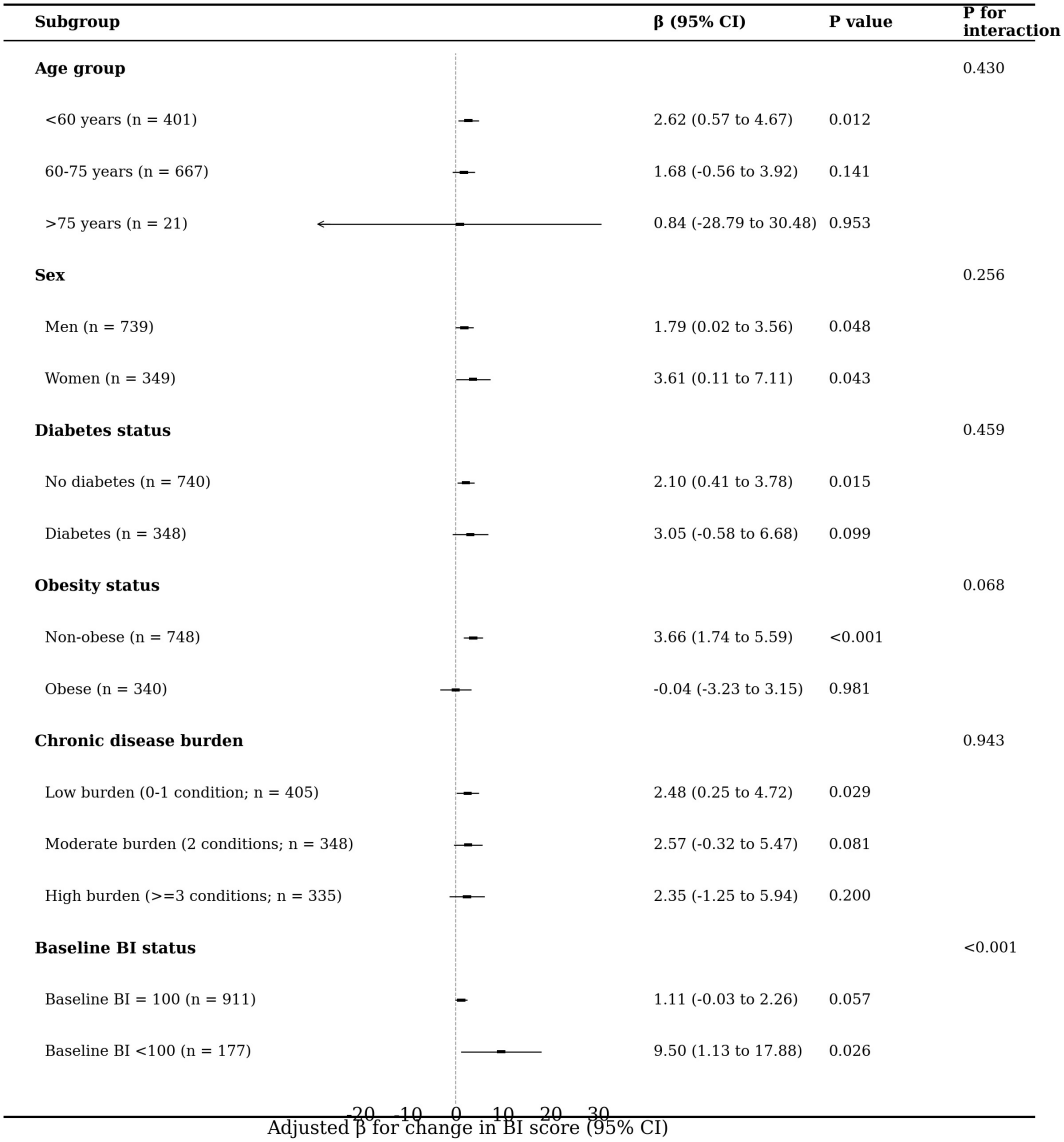
Forest plot of subgroup analyses for the association between NBP treatment and the 1-year change in Barthel Index. Squares indicate the adjusted regression coefficient (β) for NBP treatment vs. the control group within each subgroup, and horizontal lines indicate the corresponding 95% confidence interval (CI). In every subgroup the control group is the reference category, so β represents the additional change in Barthel Index score associated with NBP treatment; values to the right of the vertical line at β = 0 (the line of no effect) indicate greater improvement with NBP. Models were adjusted for the same covariates as the fully adjusted model, except for the corresponding stratification variable. *P* for interaction was derived from an interaction term between NBP treatment and each subgroup variable.

A significant interaction was observed according to baseline BI status (P for interaction < 0.001). Among patients with baseline BI < 100, NBP treatment was associated with a larger BI improvement (β = 9.50, 95% CI: 1.13 to 17.88; *P* = 0.026), whereas the association was smaller and borderline among those with baseline BI = 100 (β = 1.11, 95% CI: −0.03 to 2.26; *P* = 0.057; Table 4; Figure 2). A baseline Barthel Index of 100 was recorded in 436 of 533 NBP-treated participants (81.8%) and 481 of 562 control participants (85.6%; *P* = 0.106).

**Table 4:** Subgroup analyses of the association between NBP treatment and 1-year BI improvement.

Subgroup	β (95% CI)	*P* value	*P* for interaction
Age group			0.430
< 60 years (*n* = 401)	2.62 (0.57 to 4.67)	0.012	
60–75 years (*n* = 667)	1.68 (−0.56 to 3.92)	0.141	
>75 years (*n* = 21)	0.84 (−28.79 to 30.48)	0.953	
Sex			0.256
Men (*n* = 739)	1.79 (0.02 to 3.56)	0.048	
Women (*n* = 349)	3.61 (0.11 to 7.11)	0.043	
Diabetes status			0.459
No diabetes (*n* = 740)	2.10 (0.41 to 3.78)	0.015	
Diabetes (*n* = 348)	3.05 (−0.58 to 6.68)	0.099	
Obesity status			0.068
Non-obese (*n* = 748)	3.66 (1.74 to 5.59)	< 0.001	
Obese (*n* = 340)	−0.04 (−3.23 to 3.15)	0.981	
Chronic disease burden			0.943
Low burden (0–1 condition; *n* = 405)	2.48 (0.25 to 4.72)	0.029	
Moderate burden (2 conditions; *n* = 348)	2.57 (−0.32 to 5.47)	0.081	
High burden (>=3 conditions; *n* = 335)	2.35 (−1.25 to 5.94)	0.200	
Baseline BI status			< 0.001
Baseline BI = 100 (*n* = 911)	1.11 (−0.03 to 2.26)	0.057	
Baseline BI < 100 (*n* = 177)	9.50 (1.13 to 17.88)	0.026	

Subgroup analyses used change in BI score as the dependent variable. Models were adjusted for the same covariates as the fully adjusted model, except for the corresponding stratification variable where appropriate. Interaction terms between NBP treatment and each subgroup variable were used to calculate *P* values for interaction.

### 3.7 Sensitivity analyses

In the IPTW analysis, the association between NBP treatment and BI improvement remained statistically significant (β = 2.36, 95% CI: 0.54 to 4.17; *P* = 0.011). NBP treatment was not significantly associated with change in mRS score after IPTW adjustment (β = 0.090, 95% CI: −0.037 to 0.217; *P* = 0.166).

In the conservative death-imputation analysis, patients who died before the 1-year assessment were assigned BI = 0 and mRS = 6. Under this conservative assumption, the association between NBP treatment and BI improvement remained significant (β = 2.72, 95% CI: 0.73 to 4.70; *P* = 0.007). In the fully adjusted death-imputation analysis, the association also remained significant (β = 2.83, 95% CI: 0.91 to 4.76; *P* = 0.004; Supplementary Table 4).

In the multiple imputation sensitivity analysis, the association between NBP treatment and greater BI improvement remained statistically significant in the fully adjusted model (β = 1.79, 95% CI: 0.21 to 3.36; *P* = 0.026). NBP treatment was not significantly associated with change in mRS score in the fully adjusted imputed model (β = 0.096, 95% CI: −0.013 to 0.206; *P* = 0.084; Supplementary Table 4).

**Figure 3:**
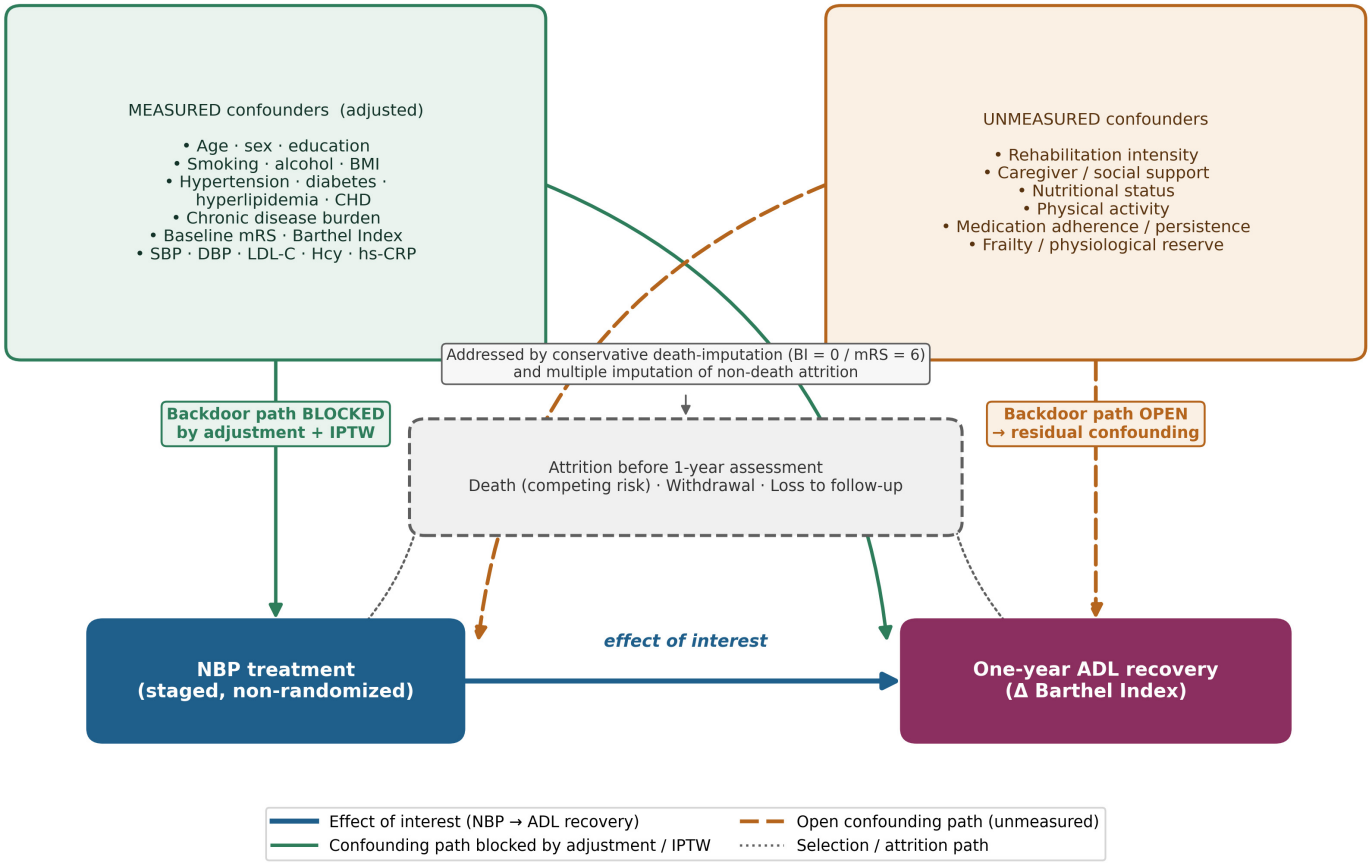
Causal framework (directed acyclic graph) for the association between NBP treatment and 1-year activities-of-daily-living recovery. The exposure (NBP treatment, allocated by a staged non-randomized strategy) and the outcome (1-year change in Barthel Index) are connected by the effect of interest. Measured confounders (demographic, lifestyle, vascular-metabolic, baseline functional, blood-pressure, and laboratory variables) open a backdoor path that was blocked by multivariable adjustment and inverse probability of treatment weighting. Unmeasured confounders (rehabilitation intensity, caregiver and social support, nutritional status, physical activity, medication adherence and persistence, and frailty) leave a residual backdoor path open, which is why the findings are interpreted as an association rather than proof of causation. Attrition before the 1-year assessment — death (a competing risk handled by conservative death-imputation) and non-death withdrawal or loss to follow-up (handled by multiple imputation) — is shown as a selection path. mRS, modified Rankin Scale; CHD, coronary heart disease; SBP/DBP, systolic/diastolic blood pressure; LDL-C, low-density lipoprotein cholesterol; Hcy, homocysteine; hs-CRP, high-sensitivity C-reactive protein; LTFU, loss to follow-up.

The assumed causal structure linking NBP treatment, measured and unmeasured confounders, selection due to attrition, and 1-year ADL recovery is summarized as a directed acyclic graph (Figure 3), which guided the selection of covariates for confounding control.

## 4 Discussion

In this post-marketing real-world non-randomized controlled study, we evaluated the association between NBP treatment and 1-year ADL recovery in patients with convalescent IS and chronic disease burden. The main finding was that NBP treatment was associated with better preservation or recovery of ADL over 1 year, as reflected by a significantly greater improvement in BI score compared with basic treatment alone. This association remained robust after multivariable adjustment, IPTW analysis, and conservative death-imputation analysis, supporting the consistency of the primary finding despite the observational nature of the study. The functional association observed in this study was specific to ADL recovery rather than global disability change. Although patients receiving NBP showed greater improvement in BI score, changes in mRS score and the proportion of patients with mRS >2 at 1 year did not differ significantly between groups. Similarly, NBP treatment was not independently associated with change in mRS score in any regression model. These findings suggest that BI may be more sensitive than mRS for detecting modest but clinically meaningful improvements in daily functional independence among convalescent stroke patients. Item-level analyses further clarified the pattern of ADL recovery associated with NBP treatment. The improvement was mainly concentrated in self-care and mobility-related activities, including grooming, toilet use, feeding, transfers, mobility on level surfaces, dressing, and stair climbing, whereas no significant differences were observed in bowel control, bladder control, or bathing. This domain-specific pattern indicates that the potential functional benefit of NBP may be more closely related to recovery or preservation of practical motor and self-care capacity than to uniform improvement across all ADL components. Subgroup analyses showed that the association between NBP treatment and BI improvement was generally consistent across clinically relevant strata, with no significant interactions by age, sex, diabetes status, obesity status, or chronic disease burden. This association is significant among non-diabetic patients, non-obese patients, and those with a lower burden of chronic diseases; however, since the interaction test did not reach statistical significance, these subgroup differences should be interpreted with caution. Therefore, the observed results in non-diabetic patients, non-obese patients, and those with a lower burden of chronic diseases should not be interpreted as evidence of differing therapeutic effects. Notably, baseline BI status significantly modified the association: patients with baseline BI < 100 showed a larger BI improvement than those with baseline BI = 100, suggesting that patients with pre-existing ADL limitation may have greater measurable potential for functional recovery.

## 5 Comparison with previous studies

The functional benefit of NBP in IS has been increasingly supported, but most previous evidence has focused on acute-stage neurological improvement rather than ADL recovery during the convalescent stage. Previous randomized trials and meta-analyses showed that NBP improved short-term neurological deficits and functional outcomes in acute IS, including reductions in NIHSS or mRS scores and increases in BI scores (7–9). Our previous work from the same community-based platform further showed that NBP reduced recurrent IS and total stroke events over 12 months, but functional outcomes were not the primary focus of that analysis (10). In contrast, the present study specifically used 1-year BI change as the primary outcome and demonstrated that NBP treatment was associated with better ADL recovery in convalescent IS patients. This finding extends prior evidence by suggesting that the potential benefit of NBP may not be limited to early neurological improvement or recurrence prevention, but may also be reflected in practical daily functional recovery during long-term community-based management. The smaller magnitude of BI change in our study compared with acute-stage trials is clinically plausible, because patients in the convalescent stage often have higher baseline BI scores and less room for measurable improvement.

The discrepancy between BI improvement and non-significant mRS change remains an important and clinically meaningful finding. It is well recognized that mRS and BI are related but not interchangeable measures of post-stroke disability. The mRS provides a global ordinal assessment of disability, whereas BI captures basic self-care and mobility-related ADL in greater detail (18). Previous clinimetric studies have shown that BI may be useful for longitudinal assessment of post-stroke functional recovery and may detect changes in functional independence that do not necessarily translate into a shift across mRS categories (18, 19). The Stroke Impact Scale and other multidomain tools were also developed because global scales alone may not fully reflect patient-centered recovery across daily activities, mobility, participation, and quality of life (20). Additional clinimetric evidence supports the value of modified BI approaches for improving sensitivity to functional change after stroke (21). Consistent with these observations, our study found that NBP treatment was significantly associated with BI improvement but not with change in mRS score or mRS >2 at 1 year. This suggests that ADL-based measures may be more sensitive for identifying modest but meaningful functional gains in a convalescent community population, particularly when baseline disability is mild to moderate.

The domain-specific pattern of ADL recovery observed in our study has rarely been described in previous NBP studies. Most clinical studies of NBP reported total BI scores, NIHSS, mRS, recurrence, or cognitive outcomes, but did not analyze individual BI components (7–12). However, post-stroke recovery is multidimensional, and improvement in ADL depends on the recovery of motor control, balance, endurance, cognition, and repeated task performance. Stroke rehabilitation guidelines emphasize that recovery requires coordinated efforts targeting mobility, self-care, daily task performance, and community reintegration (22). A clinical summary of adult stroke rehabilitation guidelines similarly highlighted that post-stroke care should extend beyond acute treatment and focus on long-term functional recovery and reintegration into daily life (23). In our study, NBP-associated BI improvement was mainly concentrated in grooming, toilet use, feeding, transfers, mobility on level surfaces, dressing, and stair climbing, whereas bowel control, bladder control, and bathing did not differ significantly between groups. This pattern suggests that the observed benefit was more closely related to self-care and mobility-related functional capacity than to all ADL domains equally. The absence of a significant effect on continence-related items may reflect different biological and clinical determinants, such as autonomic dysfunction, lesion location, aging-related urinary problems, or pre-existing urological conditions, which may be less responsive to neurovascular protective treatment.

The relationship between chronic disease burden and post-stroke functional recovery is well established, but whether NBP-related ADL benefit persists in patients with multimorbidity has remained uncertain. A systematic review and meta-analysis showed that multiple chronic conditions were associated with worse functional outcomes after IS (24). A prospective cohort study further developed a multiple chronic condition index and confirmed that chronic disease burden predicted 90-day functional outcome after IS (25). In older adults, multimorbidity combinations involving vascular, metabolic, cognitive, and musculoskeletal conditions have been associated with greater ADL and IADL disability (26, 27). Evidence from Chinese older adults also showed that chronic disease and multimorbidity were closely related to impaired daily living ability, supporting the relevance of chronic disease burden to functional vulnerability in aging populations (28). Frailty has also been linked to poorer functional outcomes and increased mortality after stroke (29). In our study, the NBP group had a higher chronic disease burden and slightly worse baseline functional status than the control group, yet NBP treatment remained associated with greater BI improvement after multivariable adjustment. Subgroup analyses further showed no significant interaction by chronic disease burden. These findings suggest that the association between NBP and ADL recovery was not limited to patients with a lower comorbidity burden. A possible explanation is that NBP has multitarget biological effects, including improvement of microcirculation, mitochondrial protection, anti-oxidative effects, anti-inflammatory effects, inhibition of apoptosis, and neurovascular repair, which may remain relevant across heterogeneous vascular-metabolic backgrounds (6).

The subgroup findings should be interpreted as exploratory, but they provide clinically useful information for future research. The association between NBP and BI improvement was observed across age and sex strata, and no significant interactions were found for age, sex, diabetes status, obesity status, or chronic disease burden. This is consistent with the concept that post-stroke functional recovery is influenced by multiple overlapping factors rather than a single comorbidity or demographic characteristic. However, the association appeared more evident in patients without diabetes, non-obese patients, and those with lower chronic disease burden, although interaction tests were not statistically significant. Previous studies have suggested that diabetes, obesity, systemic inflammation, and glycemic dysregulation may impair vascular repair, neuroplasticity, and functional recovery after stroke (12, 27). Therefore, the attenuated association in metabolically vulnerable subgroups may reflect reduced biological reserve, more severe vascular injury, or competing mechanisms of functional decline. These findings support the need for future studies to examine whether optimization of metabolic status enhances the functional benefit of NBP in convalescent stroke patients.

A particularly notable finding was the significant interaction by baseline BI status. Patients with baseline BI < 100 appeared to have a larger measurable BI improvement than those with baseline BI = 100. This is consistent with the measurement characteristics of BI. Patients with full baseline BI scores have limited room for additional improvement, resulting in a ceiling effect, whereas those with residual ADL limitation have greater potential for measurable recovery (19, 21). From a clinical perspective, this finding suggests that patients with incomplete ADL independence at baseline may represent a more informative target population for evaluating functional recovery interventions. It also supports the recommendation from stroke recovery research frameworks that outcome measures should be selected according to baseline impairment severity, timing after stroke, and expected recovery domains (30). This model also suggests that the observed association is not primarily driven by the mean-reduction effect. If the mean-reduction effect were the dominant mechanism, a larger effect would be expected in the baseline BI = 100 group (due to greater measurement error caused by the ceiling effect). However, the actual results show significantly larger treatment effects in the baseline BI < 100 group (i.e., those with residual dysfunction), supporting genuine functional benefits from NBP therapy rather than statistical artifacts. Compared to the overall effect (β = 2.40), the larger treatment effect observed in patients with a baseline Barthel Index < 100 (β = 9.50) is clinically interpretable. A difference of 9.5 points on the Barthel Index corresponds to the restoration of independence in 1–2 functional activities, representing a meaningful transition from partial dependence to functional independence for patients with baseline dysfunction. This substantial subgroup effect likely reflects that the overall estimate was diluted by the ceiling effect in patients with a baseline BI of 100, rather than a statistical artifact; the significant interaction effect (P < 0.001) supports this interpretation. However, due to the small sample size in this subgroup, the results should be interpreted with caution, and future studies should validate the specific benefits of NBP in patients with baseline dysfunction across larger cohorts.

The robustness of our findings is strengthened by sensitivity analyses, but causal interpretation remains cautious. In observational studies, treatment-selection bias and residual confounding are unavoidable concerns. The use of IPTW is an established strategy to improve baseline comparability and reduce measured confounding in non-randomized treatment comparisons (16, 17). In the present study, the association between NBP treatment and BI improvement remained significant after IPTW adjustment and conservative death imputation. By contrast, the association with mRS change remained non-significant, reinforcing the consistency of the ADL-specific finding. These results suggest that the primary conclusion was not driven solely by baseline imbalance or exclusion of patients who died before follow-up. Nevertheless, because unmeasured factors such as rehabilitation intensity, caregiver support, nutrition, physical activity, and medication adherence may also influence ADL recovery, the findings should be interpreted as an association rather than proof of causality. Future multicenter randomized or pragmatic trials are needed to confirm whether NBP directly improves ADL recovery in convalescent IS patients with chronic disease burden.

Several limitations should be acknowledged. First, this study is a non-randomized real-world controlled study with non-randomized treatment assignment, necessitating cautious interpretation of causal relationships. The NBP group exhibited slightly poorer baseline functional status and higher chronic disease burden, which may introduce treatment selection bias and residual confounding. Although multivariate adjustment, subgroup analysis, IPTW (Interpolated Treatment Weighting), and conservative death-catching analysis were employed, IPTW only addresses measurable confounders; unmeasured factors may still contribute to selection bias and indication confounding and cannot be entirely eliminated. The primary analysis utilized the complete case approach, which may introduce selection bias. These findings represent only association rather than causal evidence and require further validation through randomized controlled trials. Second, this study was conducted in a single community-based population in rural northern China. This setting provides valuable real-world evidence for long-term post-stroke management outside tertiary hospitals, but it may also limit the generalizability of the findings. In particular, the high burden of vascular-metabolic comorbidity and the relatively mild baseline disability of this cohort mean that the present evidence may not extend to urban populations, tertiary-hospital cohorts, or patients with moderate-to-severe stroke. The findings apply most directly to community-dwelling, convalescent-phase ischemic stroke patients with mild residual disability and substantial chronic disease burden who are managed in primary-care or community settings. Third, although BI and mRS are widely used functional measures after stroke, they cannot fully capture all dimensions of recovery. BI focuses mainly on basic self-care and mobility-related ADL, whereas mRS reflects global disability. In this study, NBP treatment was associated with BI improvement but not with mRS change, suggesting that BI may be more sensitive to modest ADL recovery in this population. However, many patients had relatively high baseline BI scores, which may have introduced a ceiling effect and limited the measurable range of improvement, especially among patients with baseline BI = 100. Future studies should combine BI and mRS with more granular functional scales, patient-reported outcomes, quality-of-life instruments, gait or balance tests, and participation-level measures to provide a more comprehensive assessment of post-stroke recovery. Fourth, frailty was not directly assessed in this study. Although chronic disease burden and ADL limitation are closely related to functional vulnerability in older adults, they are not equivalent to frailty. Therefore, our findings should not be interpreted as evidence that NBP improves frailty itself. The absence of standardized frailty measures limited our ability to determine whether the observed ADL benefit was mediated by improved physiological reserve or reduced frailty progression. Future studies should incorporate validated frailty instruments, such as the Fried phenotype, frailty index, Clinical Frailty Scale, gait speed, grip strength, and sarcopenia-related indicators, to clarify the relationship between NBP treatment, functional resilience, and frailty-related outcomes. Fifth, changes in the Barthel Index not only reflect improvements in neurological function but may also be influenced by non-pharmacological factors such as nursing quality, rehabilitation training, and environmental adaptation; consequently, this study could not distinguish between the independent pharmacological effects of NBP and the combined effects of comprehensive management measures. Although multiple demographic, clinical, and laboratory variables were adjusted, incomplete measurements of rehabilitation outcomes and social support may have introduced residual confounding. Future research should systematically collect data on rehabilitation dosage, exercise participation, nutritional interventions, family care, and patient-reported outcomes to more accurately assess the independent contribution of NBP. Sixth, the compliance rate with NBP and changes in concomitant therapies during follow-up were not adequately characterized. Compliance in the NBP group was assessed through medication records and returned vials, but no quantitative compliance metrics were calculated, making it impossible to evaluate the association between compliance and outcomes; temporal data on concomitant therapies were also limited. If the NBP group received additional treatment optimization due to study focus, this difference would likely mitigate rather than exaggerate the observed association; thus, the current results should be regarded as conservative estimates. Future studies should incorporate electronic medication monitoring, quantitative compliance assessment, and time-varying exposure analysis. Seventh, this study did not include the National Institutes of Health Stroke Scale (NIHSS) score. Since the subjects were community-based patients with post-recovery ischemic stroke (more than 6 months after onset), all had already passed the acute phase at enrollment; consequently, the sensitivity and clinical utility of the NIHSS score in recovery-phase assessments are limited, so it was not included as a routine measurement parameter. Moreover, standardized NIHSS assessment is challenging to perform under community healthcare conditions. Therefore, we could not incorporate stroke severity as a covariate into the analysis nor rule out its potential impact on functional recovery outcomes. Future studies should record NIHSS scores during the acute phase and evaluate their predictive value for ADL recovery during the recovery phase to better control for the confounding effect of stroke severity. Additionally, the primary analysis employed complete-case analysis, excluding patients who were lost to follow-up, withdrew, died, or had incomplete 1-year functional assessments. Selection bias may arise if the treatment effect differed systematically between completers and non-completers. Although conservative death-imputation and multiple imputation sensitivity analyses were conducted, unmeasured factors that simultaneously influence the risk of attrition and functional trajectories may violate the missing-at-random assumption, resulting in residual selection bias.

Finally, this analysis focused on 1-year ADL recovery and did not fully evaluate longer-term functional trajectories or post-discontinuation effects. Functional recovery after IS may continue beyond 1 year, particularly in patients with chronic disease burden or residual ADL limitation. In addition, this study did not integrate recurrent vascular events, cognitive changes, carotid ultrasound progression, and laboratory changes into a unified recovery model. Future analyses with repeated functional assessments at 3, 6, 9, 12, 18, and 24 months may help clarify whether the observed ADL benefit is sustained after NBP discontinuation and whether it is related to recurrence prevention, vascular structural changes, or cognitive preservation.

## 6 Conclusion

In this post-marketing real-world non-randomized controlled study, NBP treatment was associated with better 1-year ADL recovery in patients with convalescent IS and chronic disease burden, mainly reflected by greater BI improvement and lower adjusted odds of ADL dependency. In contrast, changes in mRS were not significant, suggesting that ADL-based outcomes may capture meaningful recovery in daily independence that is not fully reflected by global disability scales. From the perspective of geriatric and chronic disease management, these findings extend the evaluation of NBP from recurrent vascular events and cognition to functional capacity in daily life. The association was generally consistent across chronic disease burden strata, although the clearest benefit was observed in patients with lower chronic disease burden and in those with baseline ADL limitation. This suggests that earlier functional intervention, before the accumulation of more complex multimorbidity, may be particularly relevant for preserving post-stroke independence.

Overall, this study supports the incorporation of ADL-centered outcomes into long-term post-stroke care for patients with vascular-metabolic comorbidities. Further multicenter randomized or pragmatic studies are needed to confirm these findings and clarify how NBP may be integrated into community-based strategies aimed at maintaining functional resilience and reducing care burden in aging stroke populations.

## Data Availability

The raw data supporting the conclusions of this article will be made available by the authors, without undue reservation.
