# Supplementary material for: Association of N-butylphthalide treatment with 1-year activities-of-daily-living recovery in convalescent ischemic stroke patients with chronic disease burden: a post-marketing real-world non-randomized controlled study
Source: Front Med (Lausanne). 2026 Aug 3;13:1905628. doi: 10.3389/fmed.2026.1905628 (PMC13477979; doi:10.3389/fmed.2026.1905628)
Supplement: Supplementary file 1 [file Table_1.docx]

**Supplementary Table 1. Comparison of baseline characteristics between participants included in and excluded from the one-year functional recovery analysis.**

| **Characteristic** | **Included (n = 1,095)** | **Excluded (n = 95)** | **P value** |
| --- | --- | --- | --- |
| NBP group, n (%) | 533 (48.7) | 63 (66.3) | 0.001 |
| Age, years | 62.42 (8.46) | 64.31 (9.54) | 0.065 |
| Men, n (%) | 741 (67.7) | 58 (61.1) | 0.229 |
| Education, years | 6.76 (3.57) | 6.40 (3.73) | 0.363 |
| BMI, kg/m² | 26.56 (3.56) | 26.10 (3.46) | 0.223 |
| SBP, mmHg | 155.19 (21.68) | 155.95 (23.20) | 0.759 |
| DBP, mmHg | 95.16 (12.18) | 94.46 (13.41) | 0.627 |
| Hypertension, n (%) | 825 (75.3) | 72 (75.8) | 1.000 |
| Diabetes, n (%) | 351 (32.1) | 30 (31.6) | 1.000 |
| Hyperlipidemia, n (%) | 470 (42.9) | 36 (37.9) | 0.399 |
| Coronary heart disease, n (%) | 143 (13.1) | 12 (12.6) | 1.000 |
| Obesity, n (%) | 344 (31.4) | 27 (28.4) | 0.625 |
| Chronic disease burden score | 1.95 (1.13) | 1.86 (1.20) | 0.508 |
| Baseline mRS | 1.17 (1.17) | 1.64 (1.54) | 0.004 |
| Baseline Barthel Index | 94.49 (15.92) | 86.05 (27.89) | 0.004 |

Values are mean (SD) or n (%) unless otherwise indicated. Chronic disease burden score was calculated as the cumulative number of hypertension, diabetes, hyperlipidemia, obesity, and coronary heart disease. BMI, body mass index; SBP, systolic blood pressure; DBP, diastolic blood pressure.

**Supplementary Table 2. Item-level changes in BI components according to treatment group**

| **BI component** | **NBP group**  **(n = 533)** | **Control group**  **(n = 562)** | **P value** |
| --- | --- | --- | --- |
| Bowel control | -0.03 (1.24) | -0.02 (1.66) | 0.907 |
| Bladder control | 0.01 (1.32) | 0.02 (1.84) | 0.930 |
| Grooming | 0.12 (1.28) | -0.12 (1.35) | 0.0028 |
| Toilet use | 0.23 (1.93) | -0.15 (2.09) | 0.0020 |
| Feeding | 0.13 (1.59) | -0.16 (1.88) | 0.0056 |
| Transfers | 0.23 (2.28) | -0.10 (2.52) | 0.026 |
| Mobility on level surfaces | 0.15 (2.43) | -0.21 (2.61) | 0.017 |
| Dressing | 0.17 (2.27) | -0.18 (2.15) | 0.0095 |
| Stair climbing | 0.16 (2.40) | -0.21 (2.45) | 0.011 |
| Bathing | 0.15 (1.64) | 0.02 (1.61) | 0.179 |

Values are mean (SD). Each item-level change was calculated as the 1-year item score minus the baseline item score; a positive value indicates improvement.

**Supplementary Table 3. Logistic regression analyses for binary functional outcomes at 1 year**

| **Outcome** | **Model** | **OR (95% CI)** | **P value** |
| --- | --- | --- | --- |
| mRS >2 at 1 year | Unadjusted | 1.04 (0.71 to 1.53) | 0.837 |
| mRS >2 at 1 year | Fully adjusted | 0.63 (0.37 to 1.07) | 0.086 |
| BI <=60 at 1 year | Unadjusted | 0.78 (0.48 to 1.27) | 0.315 |
| BI <=60 at 1 year | Fully adjusted | 0.42 (0.22 to 0.80) | 0.008 |

Fully adjusted models included age, sex, education, smoking, alcohol use, BMI, hypertension, diabetes, hyperlipidemia, coronary heart disease, baseline mRS, baseline BI, SBP, DBP, LDL-C, homocysteine, and hs-CRP.

**Supplementary Table 4. Sensitivity analyses for the association between NBP treatment and functional recovery**

| **Sensitivity analysis** | **Outcome** | **β (95% CI)** | **P value** |
| --- | --- | --- | --- |
| IPTW analysis | Change in BI score | 2.36 (0.54 to 4.17) | 0.011 |
| IPTW analysis | Change in mRS score | 0.090 (-0.037 to 0.217) | 0.166 |
| Conservative death imputation | Change in BI score | 2.72 (0.73 to 4.70) | 0.007 |
| Conservative death imputation | Change in mRS score | 2.83 (0.91 to 4.76) | 0.004 |
| Multiple imputation | Change in BI score | 1.79 (0.21 to 3.36) | 0.026 |
| Multiple imputation | Change in mRS score | 0.096 (-0.013 to 0.206) | 0.084 |

In the IPTW analysis, inverse probability of treatment weighting was applied using propensity scores based on baseline covariates. In the conservative death imputation analysis, patients who died before the 1-year assessment were assigned BI = 0 and mRS = 6.
